# Concise Review: Stem/Progenitor Cell Proteoglycans Decorated with 7‐D‐4, 4‐C‐3, and 3‐B‐3(‐) Chondroitin Sulfate Motifs Are Morphogenetic Markers of Tissue Development

**DOI:** 10.1002/stem.2860

**Published:** 2018-07-31

**Authors:** Anthony J. Hayes, Susan M. Smith, Bruce Caterson, James Melrose

**Affiliations:** ^1^ Bioimaging Research Hub, Cardiff School of Biosciences Cardiff University Cardiff Wales, United Kingdom; ^2^ Raymond Purves Bone and Joint Research Laboratories Kolling Institute of Medical Research, Royal North Shore Hospital and University of Sydney St. Leonards New South Wales Australia; ^3^ School of Biosciences Cardiff University Cardiff Wales, United Kingdom; ^4^ Graduate School of Biomedical Engineering University of New South Wales Sydney New South Wales Australia

**Keywords:** Cell surface markers, Chondroitin sulfate, Developmental biology, Differentiation, Fetal stem cells, Glycosaminoglycan, Mesenchymal stem cells

## Abstract

This study reviewed the occurrence of chondroitin sulfate (CS) motifs 4‐C‐3, 7‐D‐4, and 3‐B‐3(‐), which are expressed by progenitor cells in tissues undergoing morphogenesis. These motifs have a transient early expression pattern during tissue development and also appear in mature tissues during pathological remodeling and attempted repair processes by activated adult stem cells. The CS motifs are information and recognition modules, which may regulate cellular behavior and delineate stem cell niches in developmental tissues. One of the difficulties in determining the precise role of stem cells in tissue development and repair processes is their short engraftment period and the lack of specific markers, which differentiate the activated stem cell lineages from the resident cells. The CS sulfation motifs 7‐D‐4, 4‐C‐3, and 3‐B‐3 (‐) decorate cell surface proteoglycans on activated stem/progenitor cells and appear to identify these cells in transitional areas of tissue development and in tissue repair and may be applicable to determining a more precise role for stem cells in tissue morphogenesis. stem cells *2018;36:1475–1486*

**Significance Statement**

Interest in stem cells and their therapeutic potential has exploded in recent years. Elucidation of the complexity of the carbohydrate components of the stem cell surface has yielded important information on their differentiation status and identified those cells committed to a pluripotent state. Activated stem cells differentiate along specific differentiation pathways into specific cell lineages. This is reflected in their cell surface glycodynamics and can be monitored using specific monoclonal antibodies which identify the 4C3, 7D4 and 3B3(‐) sulfation motifs on cell surface chondroitin sulfate proteoglycans. These are also useful to monitor tissue morphogenetic changes in development and tissue repair.

## Introduction

This article reviews the chondroitin sulfate (CS) sulfation motifs identified by monoclonal antibodies (mAbs) 4‐C‐3, 7‐D‐4, and 3‐B‐3 (‐), which decorate proteoglycans associated with stem/progenitor cell populations in tissue development and in pathological remodeling.

### The CS Sulfation Motif Presentations

**Figure 1:**
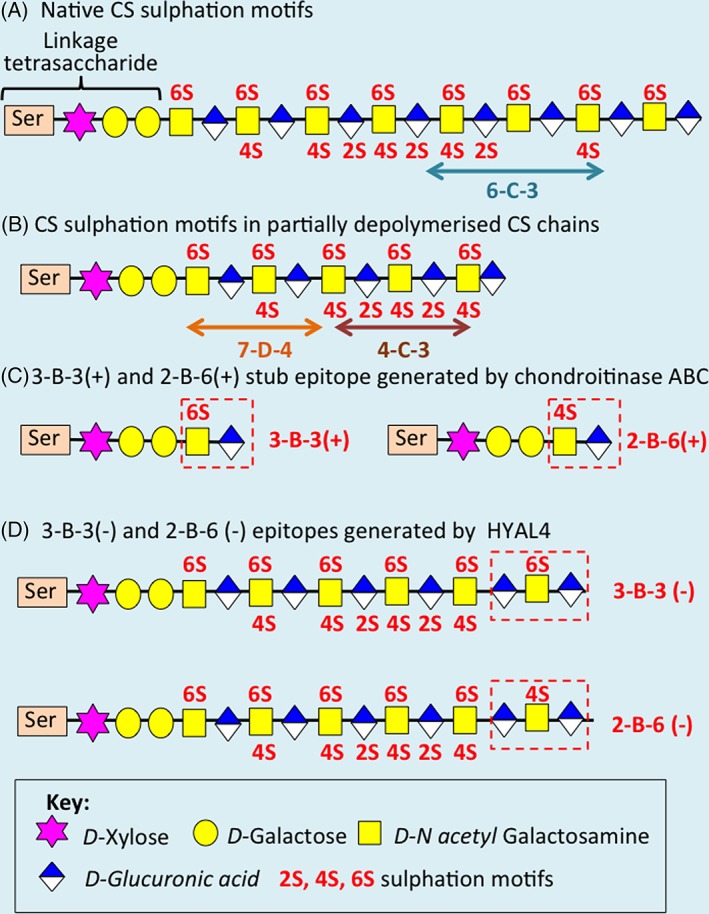
**(A):** CS sulfation motif organization in a CS chain showing the terminal 3‐B‐3 (−) motif and 6‐C‐3 motifs. **(B)**: The 7‐D‐4 and 4‐C‐3 motifs have been mapped by partial digestion of CS chains by chondroitinase ABC. **(C)**: A terminal unsaturated 3‐B‐3(+) stub epitope is also generated when the CS chain undergoes complete digestion with chondroitinase ABC. **(D)**: HYAL4 generates the 2‐B‐6(−) and 3‐B‐3(−) epitopes.

CS is a glycosaminoglycan (GAG), which is composed of β1‐3 and β 1‐4 glycosidically linked D‐glucuronic acid and N‐acetyl galactosamine *O‐*sulfated at the 2, 4, and C6 positions in a repeat disaccharide 1, 2. The D‐glucuronic acid moiety of CS may be epimerized to α *L*‐iduronic acid in the related GAG dermatan sulfate (DS). This leads to a considerable degree of structural diversity in CS/DS with more than 1,000 pentasaccharide sequences possible, which can explore a varied number of interactive structural conformations 1, 2. This large array of structures explains why these CS motifs interact with such a diverse repertoire of cytokines, chemokine's, morphogens, and growth factors, which regulate cellular differentiation and proliferation during tissue development 1, 2, 3, 4, 5. The 3‐B‐3 (−), 6‐C‐3, 7‐D‐4, and 4‐C‐3 CS sulfation motifs in CS chains are depicted in Figure 1. These were identified in intact native CS chains and following partial depolymerization using chondroitinase ABC (Fig. 1A, 1B) 1. Complete enzymatic digestion of the CS chain using chondroitinase ABC generates unsaturated 3‐B‐3 (+) and 2‐B‐6 (+) stub disaccharide epitopes (Fig. 1C). These should not be confused with the 3‐B‐3 (−) and 2‐B‐6 (−) epitopes, which are terminal epitopes generated by hyaluronidase (HYAL 4) (Fig. 1D).

The mast cell proteoglycans serglycin and perlecan have been reported to display a 2‐B‐6 (−) epitope on their CS chains 6. In similar to 3‐B‐3 (−), 2‐B‐6 (−) is not generated by chondroitinase ABC digestion. The 2‐B‐6 (−) epitope has previously been reported in osteoarthritic cartilage 7. Generation of this epitope is due to the action of a member of the HYAL family, HYAL‐1 or HYAL‐4, which are CS hydrolases cleaving CS in the β1 → 4 disaccharide glycosidic linkage 8. In addition to the generation of the 2‐B‐6(−) epitope in chondroitin‐4‐sulfate, this enzyme may also act on chondroitin‐6‐sulfate chains to generate the 3‐B‐3 (−) CS epitope. The 2‐B‐6 (−) and 3‐B‐3(−) epitopes and the production of HYAL‐4 by mast cells are associated with tissue development and with connective tissue remodeling in inflammatory conditions. The findings of this study also suggest that like mast cells, chondroprogenitor cells may express HYAL‐4 and thus generate the 3‐B‐3(−) epitope; however, this may be attached to a different proteoglycan to that found in mast cells.

### Raising of CS Sulfation Motif Antibodies and Identification of the Epitopes They Identify in Connective Tissues

In the early 1980’s, a range of anti‐CS mAbs were developed and shown to recognize specific sulfation motif epitopes in cell and matrix GAGs . Initial expectations were that mAbs against GAG epitopes would be extremely difficult to raise as these represent highly conserved structures occurring throughout invertebrate and vertebrate evolution more than hundreds of millions of years. However, with the use of a hyperimmunization protocol and draining lymph nodes as the source of lymphocytes for hybridoma production, a large number of mAbs recognizing highly conserved protein and carbohydrate epitopes on macromolecules were developed and shown to be applicable to the investigation of a wide variety of animal species 9, 10, 11. These mAbs recognized different CS isomer neoepitopes and native CS GAG chains 1, 9, 11, 12, 13. Then, two of these new mAbs (6‐C‐3 and 7‐D‐4) were used in immunohistochemical studies examining the location of different CS‐proteoglycans in developing Bursae of Fabricius isolated from embryonic chickens 9. This study clearly demonstrated differential staining patterns of CS sulfation motifs on cell surface and matrix proteoglycans during embryonic chick lymphopoiesis. Retrospectively, and more recently, analysis of this data suggested that these differential staining patterns were present on different stem, progenitor, and stromal cells undergoing different stages of lymphopoiesis in the developing Bursa.

### Use of CS‐Sulfation Motif Antibodies in Immunolocalization Studies

**Figure 2:**
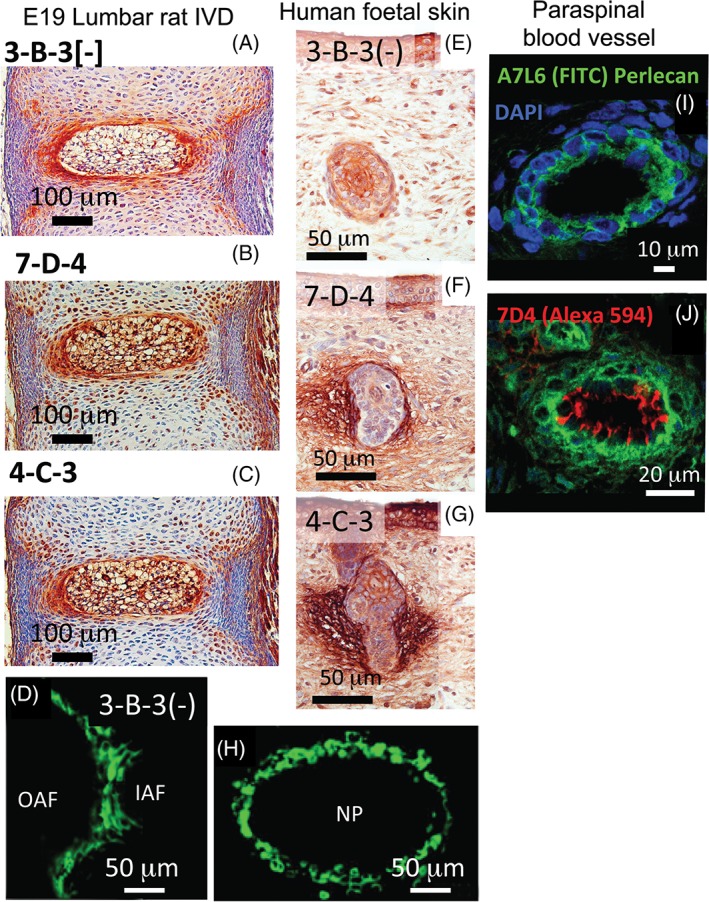
(**A**), (**B**), (**C**) Immunoperoxidase localisation of CS sulphation motif epitopes 3B3(‐), 7D4 and 4‐C‐3, respectively, in a lumbar rat intervertebral disc at E19. (D), (F) Immunofluorescence localisation of 7D4 and 3B3(‐)in transitional tissues of the lumbar rat E19 disc. (E), (F), (G) Immunoperoxidase localisation of 3B3(‐), 7D4 and 4‐C‐3 in 14 week old gestational age human foetal skin. Note differential staining of CS motifs around hair follicles. (I), (J) Immunofluorescence localisation of perlecan (green) and the 7D4 CS sulphation motif epitope (red) in human paraspinal blood vessels at 14 week gestational age (DAPI counterstained nuclei shown in blue). Scalebars in microns. Figure segments (A–C) reproduced from reference [14], (D–E) from reference [22] and (E–G, I, J) from [15] with permission. Figure segments (I) and (J) were originally published in The Biochemical Journal. Hayes A, Sugahara K, Farrugia B, Whitelock JM, Caterson B, Melrose J. Biodiversity of CS‐proteoglycan sulfation motifs: chemical messenger recognition modules with roles in information transfer, control of cellular behaviour and tissue morphogenesis 20186 475 [3]:587–620 © copyright Biochemical Society.

**Figure 3:**
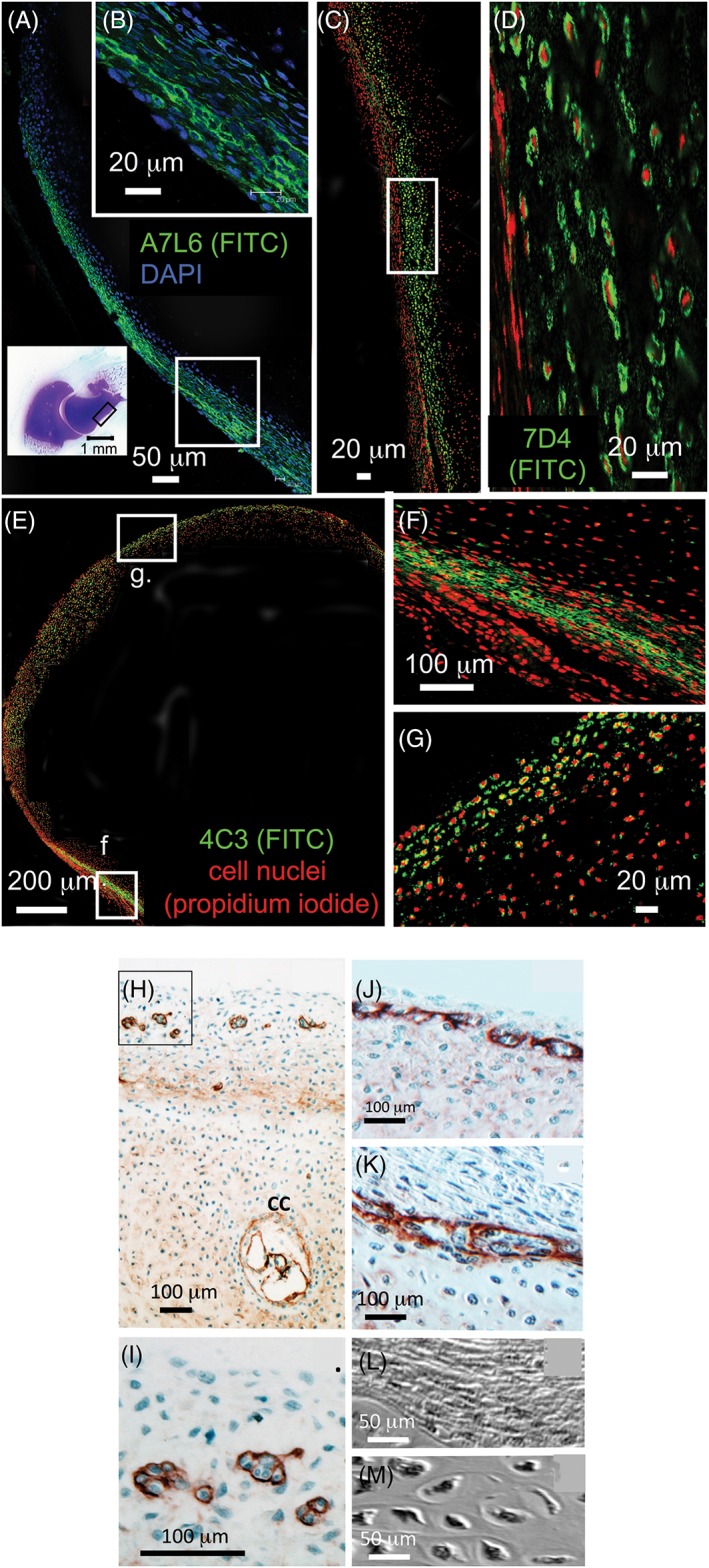
(**A**) Confocal fluorescence image of a 14 week‐old gestational age human fetal elbow depicting perichondrial perlecan labelling (green) immunolocalized with a perlecan domain IV mAb (A7L6). The localization of the A7L6 primary antibody was visualized using a Fluorescein isothiocyanate (FITC)‐labeled secondary antibody, cell nuclei were counterstained with DAPI (blue). Bottom left inset shows the whole elbow joint after toluidine blue staining for total sulphated proteoglycan. Boxed area in bottom right of micrograph shows perichondrial region examined at higher magnification in B (inset, top right). (**B**) perlecan labelling delineates putative stem cell niches in high power magnification of perichondrial region. Figure reproduced from [16] with permission. (**C**) Confocal fluorescence image shows that labelling of the 7‐D‐4 CS sulfation motif is also associated with transitional perichondrial tissues (green labelling). The area in (**C**) is depicted at higher magnification in (**D**). The 7‐D‐4 epitope was visualized using a FITC‐labeled secondary antibody. Cell nuclei were counterstained with propidium iodide (red). Figure reproduced from [16] with permission. (**E**), (**F**), (**G**) Confocal fluorescence images showing labeling of the 4‐C‐3 CS sulfation motif epitope in the surface regions of the developmental elbow (**E**, **G**) and perichondrium (**F**). Boxed area in (**E**) shown at higher magnification in (**G**). The 4‐C‐3 epitope was visualized using a FITC‐labeled secondary antibody, cell nuclei were counterstained with propidium iodide (red). Figure reproduced from [16] with permission. (**H**), (**I**), (**J**) Immunolocalization of perlecan in tibial cartilage from a 14 week‐old human fetal hip (**H**), (**I**) and knee (**J**), (**K**) delineating small discrete groups of cells in stem cell niches within the rudiments. The cellular morphologies in the stromal tissue associated with the rudiment surface regions (**L**) and within the rudiment proper (**M**) differed markedly in non‐stained Nomarsky DIC images. Figure modified from [100] with permission. Perlecan immunolocalization using a domain IV perlecan antibody (mAb A7L6) and alkaline phosphatase‐labelled secondary antibody using NovaRED for visualization.

The development of mAbs to specific CS sulfation motifs (Fig. 1, Table 1) has facilitated the identification/immunolocalization of specific subsets of cells that reside in morphological zones, where stem/progenitor cells are found in musculoskeletal tissues including articular cartilage, tendons, and intervertebral disc (IVD) (Fig. 2A–2D). These mAbs also identify subsets of cells in morphological zones from several other tissues where stem/progenitor cells reside; that is, the crypts of the gut villae (mouse) and the limbus at the interface of the cornea and the sclera in the developing chick eye, and subsets of cellular zones in the developing chick bursa where hematopoiesis and lymphopoiesis occur 1, 9, 10, 11, 12, the hair follicle and skin (Fig. 2E, 2G)). CS sulfation motifs occur at many important centers/interfaces of growth and differentiation in the development of a wide range of connective tissues. During embryonic development of the IVD of the rat spine, the CS motif recognized by mAb 7‐D‐4 is associated with cells of the transitional growth tissue that occurs between the inner and outer annulus, which acts as a physis in the IVD 14. Furthermore, the GAG epitope recognized by mAb 3‐B‐3 (−) is associated with a specific subpopulation of cells surrounding the developing nucleus pulposus (Fig. 2H). CS sulfation motifs 3‐B‐3 (−), 7‐D‐4, 4‐C‐3, and 6‐C‐3 are also differentially expressed in developing tendon; limbus; skin, gut, and articular cartilage, all occurring at important sites of tissue differentiation and growth/renewal. In articular cartilage, the differential distribution of these CS sulfation motifs is, however, particularly intriguing (Figs. 3C, 3G). Here, 3‐B‐3 (−), 7‐D‐4, and 4‐C‐3, are associated with specific subpopulations of chondrocytes within the superficial zone of the tissue following joint cavitation: 3‐B‐3(−) is expressed by a small subset of chondrocytes at the surface; 7‐D‐4 is expressed by all superficial zone cells to a depth of 2–3 cells; and 4‐C‐3 reactivity is more widespread, extending to a slightly greater depth within the tissue. This zone not only contains a chondroprogenitor cell population, but growth factors of the TGF‐β and IGF families are also sequestered here during development 17, 18, 19. Discrete small niches are also surrounded by perlecan in the surface regions of hip, knee, and elbow cartilage rudiments (Fig. 3H) 20 and also in a perichondrial region of the elbow (Fig. 3F). Similar stem cell niches have also been demonstrated in the outer AF‐vertebral growth plate margins in a number of species 21 and in peripheral regions of the nucleus pulposus (NP) in the developmental rat IVD (Fig. 2A, 2C)) 22. Perlecan associated with such niches has been shown to be a hybrid proteoglycan substituted with HS and the 7‐D‐4 CS motif 21. Immunolocalization of perlecan in human fetal knee and hip cartilage is a useful technique to delineate the stem cell niches where the progenitor cells reside in the surface regions of these cartilages (Fig. 3H, 3K)). Recently, cell nests have been identified in adult ovine IVD tissues and shown to be surrounded by a dense layer of hyaluronan (HA). HA has also been described surrounding stem cell niches where it has a role in the maintenance of stem cells in a quiescent state of self‐renewal 23, 24. Similar cell nests have also been identified in adult human IVDs 25, isolated, cultured, and shown to express 4‐C‐3, 7‐D‐4, and 3‐B‐3(−) CS sulfation motifs and a number of stem cell CD markers by flow cytometry . These represent adult stem cell niches in the IVD. Such cell nests also occur in grade IV degenerate human IVDs and in an ovine model of experimental disc degeneration 25.

### Evidence of Stem/Progenitor Cells Within the Surface Regions of the Cartilaginous Rudiments of Diarthrodial Joints

**Figure 4:**
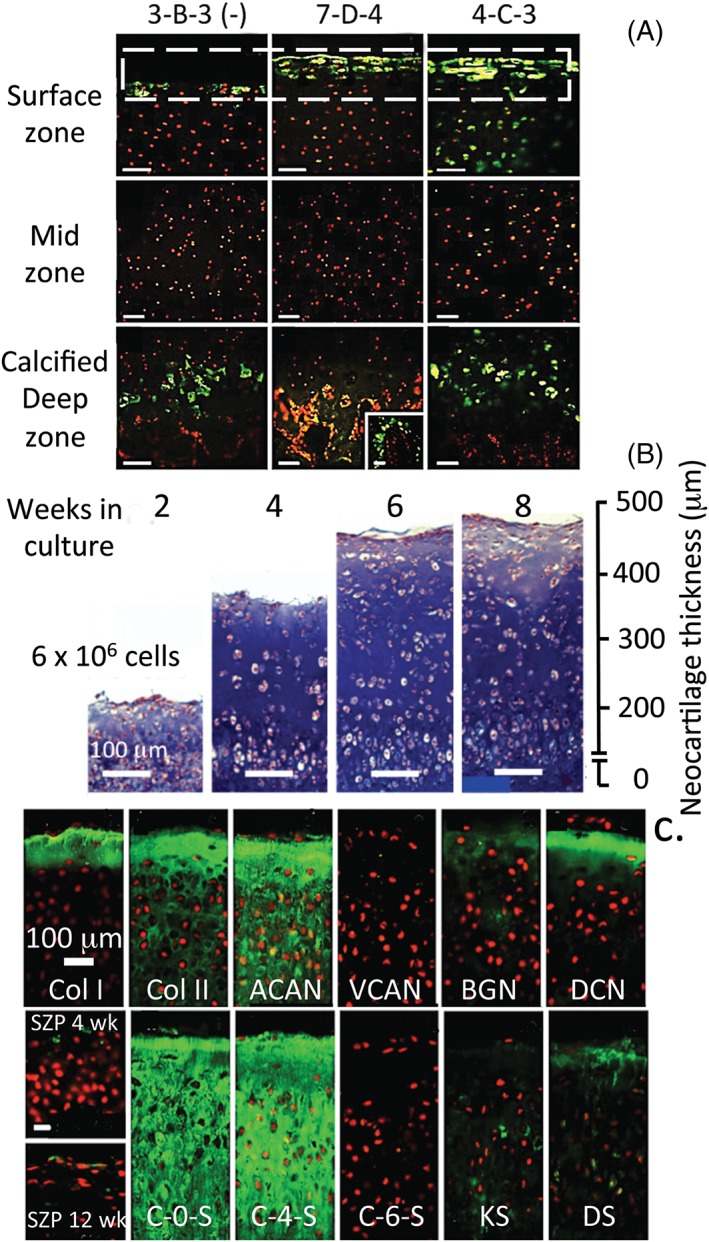
**(A)**: Composite figure showing immunofluorescent labeling patterns of the CS sulfation motifs 3‐B‐3(−), 7‐D‐4, and 4‐C‐3 in the superficial and deep regions of juvenile bovine knee articular cartilage. A stem cell progenitor cell population is present in the surface regions of developing cartilage rudiments 17, 18. **(B):** Neocartilages grown on Millipore filter membranes (6 × 10^6^ cells/insert) following 2, 4, 6, and 8 weeks culture. The increase in neocartilage thickness over the culture period is shown in the Alcian blue‐stained bright‐field images. Immunofluorescent localizations of collagen types I and II, aggrecan and versican, biglycan, decorin and lubricin shows that the neocartilage has a similar composition and organization to mature articular cartilage **(C)**. The neocartilage was particularly rich in C‐0‐S and C‐4‐S, which is typical of an immature cartilage, but contained little C‐6‐S, KS, and DS, which are associated with maturational stages of cartilage development. Figure modified from Hayes AJ, Hall A, Brown L, Tubo R, Caterson B. Macromolecular organization and in‐vitro growth characteristics of scaffold‐free neocartilage grafts. *J Histochem Cytochem.* 2007;**55** 8:853–66. DOI: 10.1369/jhc.7A7210.2007 with permission SAGE publishers.

Numerous studies have demonstrated the presence of stem/progenitor cell subpopulations within the surface regions of articular cartilage of diarthroidal joints 18, 26, 27, 28. These cells have been shown to retain their chondrogenic potential during extended monolayer culture and exhibit phenotypic plasticity in their differentiation pathway, thus may have significant potential in cell‐based articular cartilage repair therapies. Our studies show that primary chondrocytes from full depth immature bovine articular cartilage are capable of recapitulating a zonally organized neocartilage tissue when grown in vitro on MilliPore filter membranes (MilliporeSigma, Billerica, MA) (Fig. 4A, 4B) 29. The matrix composition and organization of this tissue are strikingly similar to developmentally immature articular cartilage in vivo. Neocartilages produced more than an 8 week culture period have prominent surface expression of type I collagen (Fig. 4C) with type II collagen and aggrecan throughout the cartilage depth (Fig. 4C). No expression of versican is observed, but decorin, biglycan, and lubricin occur in the surface region (Fig. 4C). Chondroitin‐0‐sulfate and C‐4‐S are also prominent CS isomers (Fig. 4C) but not chondroitin‐6‐sulfate (C‐6‐S), keratan sulfate (KS), or dermatan sulfate (DS) (Fig. 4C). These findings are typical of an immature cartilage, which does not contain appreciable levels of C‐6‐S, KS, and DS, which appear later with tissue maturation (Fig. 4B). Chondrocytes enriched from the superficial zone of articular cartilage generate similar zonally organized neocartilage when grown under identical culture conditions; however, cells enriched from mid or deep zone appear considerably less effective in this regard 30. Similar zone‐specific, maturational differences of different chondrocyte subpopulations grown in vitro have been noted by other groups 31, 32. This data suggest that superficial zone cells have a greater developmental repertoire than cells in subjacent tissue zones, consistent with appositional growth being driven by a stem/progenitor cell subpopulation resident within the superficial zone of the native tissue 18, 33. The observation that CS sulfation motifs are strongly associated with chondrocytes of the superficial zone of the native tissue suggests that these unique sulfation motifs may be associated with the distinct cell phenotypes involved in the initial stages of the chondrocyte differentiation pathway 17, 18, 29, 34, 35 (i.e., stem–progenitor—transit‐amplifying unit cell). Indeed, we have observed a high degree of similarity in the labeling patterns of both aggrecan and perlecan with mAb 4C3 suggesting that there may be CS substitution, and hence functional modification, of both aggrecan and perlecan in this superficial zone 19. The perlecan knockout mouse shows considerable cartilage pathology 36, and perlecan has well‐established roles in cartilage matrix stabilization and chondrogenesis supporting this hypothesis 37, 38. Perlecan is a hybrid HS‐CS‐proteoglycan in articular cartilage, and in the growth plate contains 4,6 disulfated CS‐E motifs that direct collagen fibrillogenesis 39.

### FGF‐18 Promotes Early Chondrogenesis and Maturational Osteogenic Differentiation of Bone Marrow Stromal Stem Cells

**Figure 5:**
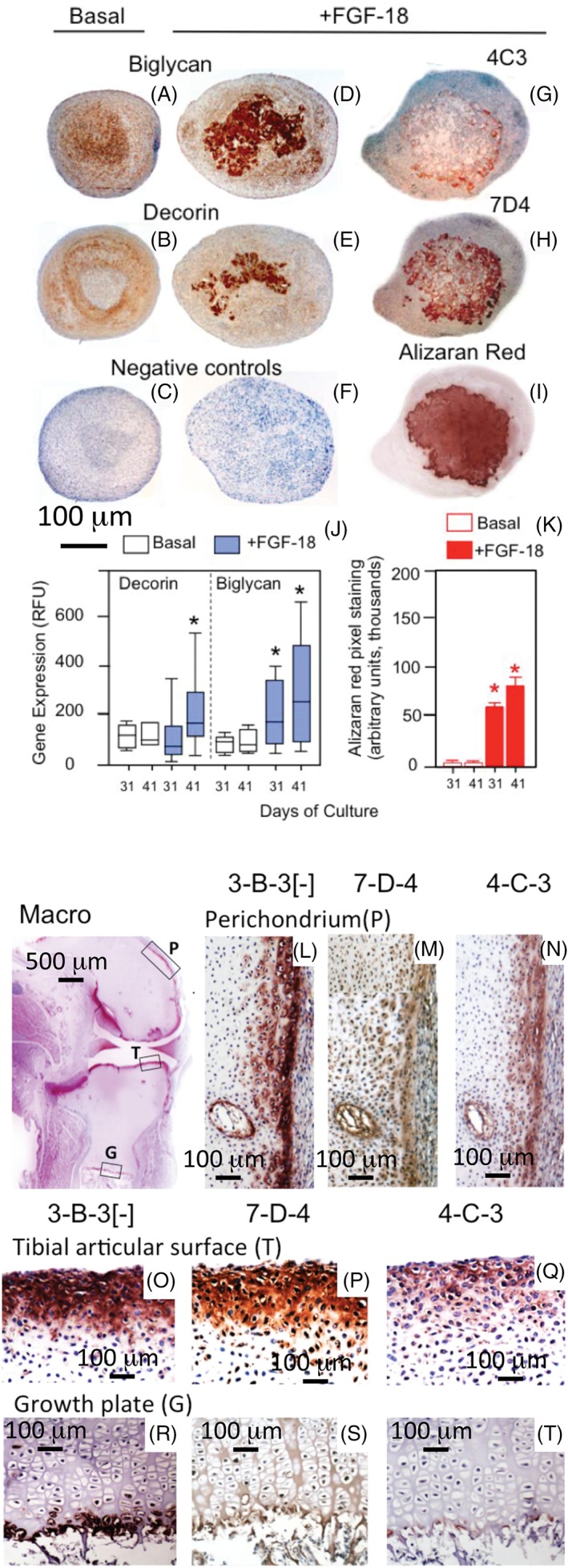
FGF‐18 promotes early chondrogenesis and maturational osteogenic differentiation of ovine stromal stem cells grown in micromass pellet culture. **(D), (E):** Decorin and biglycan were strongly upregulated by FGF‐18 on days 31–41. The CS sulfation motifs 4‐C‐3 and 7‐D‐4 **(G)**, **(H)** followed a similar deposition to that of calcium in the pellets evident by Alizaran staining **(I)**. Figure modified from 40 with permission. Histograms depict RTPCR data showing the increase in decorin and biglycan expression with FGF‐18 treatment **(J)**, and densitometric morphological data depicting Calcium deposition levels in pellets **(K)**. Immunolocalization of 3‐B‐3(−), 7‐D‐4, and 4‐C‐3 CS sulfation motifs in transitional tissues in fetal knee joint development in a 14 –week‐old gestational age human fetal knee. 3‐B‐3(−), 7‐D‐4, and 4‐C‐3 were immunolocalized in the rudiment tibial surface, perichondrium, and tibial growth plate. Figure modified from 41 with permission.

In a recent study using bone marrow stromal stem cells, FGF‐18 was shown to stimulate these cells along a chondrogenic differentiation pathway 40. FGF‐18 initially promoted chondroblasts to a committed chondrocytic phenotype and later stimulated chondrocyte maturational changes toward an osteogenic phenotype 40. Decorin and biglycan were significantly upregulated by the FGF‐18 treatment (Fig. 5D, 5E, 5J) and shown to be immunolocalized in the micro mass cell pellets in the same region where calcium deposition occurred (Fig. 5I, 5K). Consistent with our earlier findings with FGF‐18, some of the chondroprogenitor cells expressed the 4‐C‐3 and 7‐D‐4 CS sulfation motifs and these were also located in the same regions of the pellet where calcium deposition occurred (Fig. 5G, 5H). Thus, FGF‐18 promoted sequential chondrogenic commitment and an osteogenic phenotype in the stromal stem cells.

### CS Sulfation Motifs as Molecular Markers of Cell Signaling in Tissue Morphogenesis

GAG chains store and transfer information to cells providing molecular recognition and activity signals, which modulate cell growth and development by regulating growth factors such as the FGF family, Hedgehog, Wingless, and the Semaphorins 42, 43. Much progress has been made in recent years in our understanding of the contribution of GAGs to tissue development in health and ECM remodeling in disease processes. A number of publications on CS have demonstrated that these have important roles in health and disease 1, 5. Virtually, every cell produces GAGs, which are incorporated into a cell associated glycocalyx, their interactive partners and the biological processes they affect are all areas of importance in tissue development and in repair processes in tensional and weight bearing connective tissues 44. A greater understanding of these processes may improve tissue regeneration strategies. Gaining this knowledge will also provide the scientific research community with new insights as to how the pericellular environment surrounding stem/progenitor cells may regulate their senescence and subsequent activation to proliferate and differentiate into more mature cell populations during tissue growth and development and in tissue repair.

Aggrecan is the major CS‐substituted proteoglycan of cartilaginous tissues, with well‐known extracellular matrix stabilizing, space‐filling, and water imbibing properties that equip these tissues with dynamic resilience to compressive loading 44. Correct sulfation of CS‐proteoglycans is essential for proper Indian hedgehog signaling in the developing growth plate 45, perlecan, a hybrid CS‐HS proteoglycan in cartilage is also responsible for the localization and activity of the related Sonic hedgehog protein 46. Native CS sulfation motifs such as 7‐D‐4, 4‐C‐3, and 3‐B‐3(−) on proteoglycans may serve to immobilize growth factors/morphogens actively involved in hematopoiesis, skin morphogenesis, chondrogenesis, and IVD development 1, 14, 18, 21. The unique distributions of native CS sulfation motifs with surface zone progenitor cells in articular cartilage 18, 35 and strategically located within the developmental IVD 14 and human fetal elbow 16 suggests that these identify an early stage of progenitor cell differentiation 14, 18.

### Identification of Embryonic Stem Cells

Murine and human embryonic stem cell markers have been extensively documented 47, 48 and used in the determination of their pluripotent state 49, 50 and in the characterization of specific progenitor cell populations from a number of tissues 40, 51, 52, 53, 54, 55, 56, 57, 58, 59, 60. Surface stem cell markers have also facilitated the isolation of specific stem cell populations 48, 49, 50, 51, 61, 62, 63. Cell surface markers identified by mAbs (GCTM‐2, TRA‐1‐60, TRA‐1‐81, TG343, and PHM‐5) also have important roles to play in the identification of differentiation dependent post‐translational changes in cell surface carbohydrate epitopes in pluripotent stem cells, which demonstrate cell lineage commitment and have been used in the staging of MSC maturation 49. PMH‐5 is a monoclonal antibody that identifies a 140 kDa glomerular epithelial surface coat sialoglycoprotein present in the glycocalyx of podocytes, which was called as podocalyxcin 64.

Antibodies to GCTM‐2, TRA‐1‐60, TRA‐1‐81, and TG343 identify a cell surface KS‐proteoglycan with TRA‐1‐60 and TRA‐1‐81 identifying a KS side chain epitope, whereas GCTM‐2 and TG343 identify core protein epitopes. PHM‐5 identifies the transmembrane proteoglycan podocalyxcin and SSEA‐3 and SSEA‐4 identify a carbohydrate epitope in a cell surface glycolipid 48. Subsequent studies showed that TRA‐1‐60 and TRA‐1‐81 identified the KS chains of podocalyxcin, 65 whereas GCTM‐2 identified podocalyxcin core protein. Precise elucidation of the TRA‐1‐60 and TRA‐1‐81 GAG‐binding epitope by screening against a 500 GAG oligosaccharide micro‐array showed that the lactosamine oligosaccharide Galβ1‐3GlcNAcβ1‐3Galβ1‐4GlcNAc was the minimal epitope identified by this antibody, which is a core component of KS 66.

### Stem Cell Surface Carbohydrate and Protein Expression

#### The Cadherin System

The cadherin, calcium‐dependent type‐1 transmembrane proteins form adherens junctions, binding cells tightly together within tissues and have essential roles to play during embryonic development and are critical in the induction of stem cell pluripotency 67. Cell adhesion is mediated by extracellular cadherin domains, whereas intracellular cytoplasmic domains are associated with a large number of adaptor and cytoskeletal signaling proteins constituting the cadherin adhesome. The cadherin membrane‐spanning adherens junction proteins have crucial roles in cell–cell contact formation and are connected to cytoplasmic proteins, which regulate signaling pathways and relay information regarding cell interactions to the nucleus 68, 69, 70. E‐cadherin controls early differentiation of stem cells and the regulation of pluripotency. Undifferentiated embryonic stem cells express high levels of E‐cadherin and OCT‐4; however, these are lost when the cells differentiate and a switch to N‐cadherin synthesis occurs. Embryonic stem cells expressing the stem‐cell marker, stage‐specific embryonic antigen‐1 (SSEA1) also express E‐cadherin but not N‐cadherin; however when they undergo differentiation, the expression of SSEA‐1 and E‐cadherin is lost but the expression of N‐cadherin appears. E‐cadherin maintains the compact features of stem cell colonies. Interaction of ECM components with E‐cadherin can cause morphological changes in stem cells and promote their differentiation.

### Stem Cell Surface Carbohydrate Epitopes

As already indicated, niche stem cells are normally maintained in an aggregated compact form by cell surface proteins such as E‐cadherin. However, quiescent embryonic stem cells also express characteristic carbohydrate cell surface markers such as SSEA‐1, epithelial membrane core mucin antigen (ECMA)‐1 and 2, TRA 1–60 and TRA‐1‐81 (tissue rejection antigen and Trafalgar antigen), and GCTM‐2, which identify components of the large CS and KS substituted transmembrane cell surface proteoglycan podocalyxcin 71, 72. When activated, stem cells undergo differentiation and cease to express these carbohydrate markers but now express the CS‐sulfation motifs 4‐C‐3, 7‐D‐4, and 3‐B‐3(−). These sulfation motifs have yet to be specifically demonstrated as GAG components of podocalyxcin; however the importance of CS as an essential component in the attainment of stem cell pluripotency is well‐established. Therefore, the CS‐sulfation motifs usefully identify the activated stem cell phenotype during tissue morphogenesis. Antibodies that identify these specific CS sulfation motifs are therefore useful research tools, which can be used to monitor the phenotypic status of stem cells in developmental tissues.

Human embryonic stem cells display a characteristic N‐glycome consisting of a constant part and a variable portion that change during differentiation 73. Murine embryonic stem cells display complex fucosylation patterns in cell surface glycoproteins previously identified by SSEA‐1 antibody, complex fucosylation is also a characteristic glycosylation feature in undifferentiated hESC. The complexity of cell surface glycosylation patterns in the N‐glycome of human embryonic stem cells has been determined using matrix‐assisted laser desorption/ionization time‐of flight (MALDI‐TOF) mass spectrometric and NMR spectroscopic profiling 73. Lewis X antigen (Le ^*X*^) is an abundant component of these sialylated complex‐type N‐glycans and has been used previously as a marker of embryonic stem cells and multipotential cells of early embryos. Le ^*X*^ is expressed in human and murine neural stem cells 74. SSEA‐1 is also expressed specifically by developing sensory neuroblasts. Murine SSEA‐1 75 is a carbohydrate differentiation antigen of the basic structure β13 fucosyl N acetyllactosamine [Galβ1‐4 (Fucα 13)GlcNAc 15 appearing at the late 8‐cell stage of the mouse embryo 76.

### Application of Mass Spectrometry and Proteomics for the Characterization of Stem Cell Populations Through Their Expression Profiles of Cell Surface Proteins

Mass spectrometry (MS) and proteomics are powerful techniques for the comparative analysis of cellular protein expression profiles. Global approaches have been used to define the MSC proteome 77, 78 and to track differentiation dependent changes in membrane protein expression 79. Previously, comprehensive identification of specific MSC surface markers has been limited by a lack of enrichment of membrane proteins, insufficient resolution of peptides prior to MS, and inability to compare protein levels between progenitor and differentiated cell populations. The cell surface associated proteins (the surfaceome) of human bone marrow MSCs, human umbilical cord perivascular stem cells (HUCPVCs), and adult mesenchymal fibroblasts has now been undertaken in combination with subcellular protein fractionation and eight channel isobaric tagging of proteins for relative and absolute quantification (iTRAQ) to compare the proteomes of bone marrow MSCs and HUCPVCs 61. This identified 186 significantly enriched proteins in multiple cultures of MSCs and HUCPVCs compared with adult human dermal fibroblasts, and 216 proteins that were significantly downregulated. Cell‐type‐specific protein differences were also quantified. These data have allowed the construction of a protein profile repository database that enables improved selection and characterization of human mesenchymal progenitor cell populations.

### Identification of Roles for MSCs in Tissue Morphogenesis

Although mesenchymal stem/stromal cells (MSCs) are recognized as important components of the hematopoietic niche a lack of specific markers of the activated MSC phenotype has hindered the full characterization of the roles of these cells in tissue development. The subsequent identification of MSC markers such as the leptin receptor (LepR), a receptor for a fat cell‐specific hormone has been applied to fate mapping studies, which have facilitated the identification of specific MSC populations with roles in tissue morphogenesis and connective tissue remodeling 80. LepR+ cells appear postnatally in the bone marrow and are a major source of new osteoblasts and adipocytes 60. Leptin regulates bone formation 81 through differential ALK1 and ALK5 signaling by MSCs 82 regulating chondrocyte differentiation and ECM maturation during endochondral ossification 83. Leptin also increases growth of the primary ossification centers in fetal mice 84.

Immunohistochemistry of fetal cartilage tissue sections for Gremlin‐1(GREM1) and bone γ‐carboxyglutamic acid‐containing protein (BGLAP) demonstrates differential expression of these prominent MSC genes by articular chondrocytes and osteophytic chondrocytes and bone, respectively. Gremlin‐1 identifies skeletal stem cells with bone, cartilage, and reticular stromal potential 59 and is a key regulator of human articular cartilage homeostasis 85. GREM‐1 is a BMP antagonist that inhibits BMP2 and BMP4 and the TGF‐β signaling pathway during limb bud cartilage development and acts in a co‐ordinated fashion with the upregulation in FGF4 and FGF8 and SHH signaling, which occurs in cartilage development. BGLAP (osteocalcin) is a noncollagenous pro‐osteoblastic hormone, which promotes bone formation in fetal tissues.

OX‐2 glycoprotein (CD200) is a type I transmembrane glycoprotein member of the immunoglobulin superfamily, which is expressed on the cell surface of some bone marrow MSCs. Screening of human and murine bone marrow derived stem cells for cell surface markers has identified CD200 as a marker for MSCs with osteogenic potential 86 and that CD200^+^ MSCs are committed along a differentiation pathway toward an osteoblastic lineage 87.

Nestin (acronym for neuroectodermal stem cell marker) is a 240 kDa type VI intermediate filament protein which is transiently expressed during development and determines the shapes of cells through the formation of cytoskeletal microtubules 88. Nestin expression disappears with MSC differentiation and it is not detectable in adult tissues. Nestin is expressed by bone marrow MSCs 89. Interestingly, TGF‐β activates nestin‐positive MSCs to become cell clusters and inactivation of this signaling pathway reduces osteoarthritic changes in articular cartilage 90. Inhibition of TGF‐β expression by MSCs in subchondral bone attenuates OA development 90.

The identification of 4‐C‐3, 7‐D‐4, and 3‐B‐3(−) expression by joint morphogenetic cells 57 complements the aforementioned MSC markers and is a useful means of identifying a shift in cell surface marker expression in MSCs ,which have become activated and have now developed migratory properties thus can participate in tissue development and tissue morphogenesis in areas distant from the stem cell niche.

### Immunolocalization of the 4‐C‐3 and 7‐D‐4 CS sulfation Motifs Identify Chondroprogenitor Cells and Transitional Tissues Undergoing Morphogenetic Change in Foetal Joint Development Proteoglycans

The 4‐C‐3 and 7‐D‐4 CS sulfation motifs immunolocate areas of tissue undergoing morphogenesis, progenitor cells and stem cell niches in the stromal tissues adjacent to the cartilage rudiment margins and in the superficial cartilage of the articular surface zones and in the perichondrium of developing joints 16, 35. The CS sulfation motifs were also seen distally in the terminally differentiated chondrocytes of the tibial fetal growth plate in areas of cartilage calcification (Fig. 5O–5Q). Areas of the perichondrium were also prominently stained for the CS sulfation motifs, and areas of vascular ingrowth into the cartilage rudiments a few cartilage canals also were prominently stained with the 3‐B‐3(−), 7‐D‐4, and 4‐C‐3 epitopes (Fig. 5L–5N). Confocal studies on the human fetal elbow also identified prominently stained cell populations in the perichondrium and surface regions of the developing joint interzone (Fig. 3). Perlecan was prominently expressed pericellularly by somewhat flattened cells in the outer fibrous regions of the perichondrium, whereas a more rounded cell population was evident further from the rudiment surface, which had very prominent pericellular staining for the 4C3 and 7D4 CS sulfation motifs (Fig. 3D, 3F). These cells were also prominent in the surface areas of the interzone region of the developing elbow joint where the 4C3 epitope was prominently localized pericellularly 27.

### Focal Expression of CS Sulfation Motifs Identifies Stem Cells on the Luminal Surfaces of Foetal Blood Vessels in Close Proximity to Pericytes a Progenitor Cell Type for Vascular Stem Cell Development

Endothelial cells express the HS‐proteoglycan perlecan, which is a major component of vascular basement membranes. Endothelial cells focally express perlecan substituted with the 7‐D‐4 CS sulfation motif on the lumenal surface of small paraspinal blood vessels in developmental human fetal spinal tissues (Fig. 2J) 44. In mature vessels, endothelial cells normally express a monosubstituted HS‐perlecan; however, smooth muscle cells and chondrocytes express a hybrid form of perlecan containing CS and HS chains. The presence of 7‐D‐4 substituted perlecan in microvessels provides further evidence of a vessel associated progenitor cell population and a vascular stem cell niche further adding to the complexity of stem cell biology.

Pericytes are embedded in basement membrane where they communicate directly with endothelial cells in the smallest blood vessels, paracrine signaling also regulates interactions between pericytes and endothelial cells . Caplan 91 reiterated proposals made earlier by Canfield and colleagues^92^, ^93^, ^94^that pericytes had importance as a progenitor stem cell type, emphasized their perivascular origin and ubiquitous distribution in tissues throughout the human body. Pericytes have important multifunctional roles to play in the mobilization of vascular stem cells during tissue injury and also in developmental processes 95, 96 and may be of great therapeutic potential 97. This is consistent with the differentiation of cultured pericytes into multiple cell types in vitro or following transplantation 98. However, a recent cell‐lineage study showed that perivascular cells do not behave as tissue‐specific progenitors in various organs, despite their ability to differentiate into multiple cell types in‐vitro questioning the dogma proposed by Caplan that all stem cells are pericytes 99. Pericytes have roles in vascular calcification 92, display osteogenic potential 94, participate in ectopic calcification 93, establish the blood brain barrier, are sources of multipotent vascular stem cells following ischemic stroke and constitute part of a neurovascular unit required for brain development 97.

## Conclusion

CS sulfation motifs are transient, focal entities that convey subtle information to cell populations in a spatial and temporal manner, which regulate cell behavior in transitional tissues undergoing morphogenesis. Stem cells are normally protected in the niche environment and maintained in an aggregated compact form by cell surface proteins such as E‐cadherin. Embryonic stem cells when in a quiescent state also express carbohydrate cell surface markers such as SSEA‐1 , ECMA‐1 and 2, TRA 1–60 and TRA‐1‐81, and GCTM‐2, these are components of large CS and KS substituted cell surface proteoglycans 71, 72. When stem cells become activated and undergo differentiation they no longer express these markers and express many of the CS‐sulfation motifs outlined in this study, such as 4‐C‐3, 7‐D‐4, and 3‐B‐3(−). A shift from E‐cadherin expression to N‐cadherin is also evident. These changes are considered important in the attainment of pluripotency and CS has essential roles to play in these processes. The CS‐sulfation motifs are useful markers to identify the activated stem cell phenotype in tissue development involving progenitor cell populations. The antibodies raised to the CS sulfation motifs described herein are therefore useful research tools to monitor such processes.

## Author Contributions

A.J.H.: undertook the confocal immunolocalizations and contributed to manuscript writing and review. S.M.M.: undertook the bright‐field microscopy and aided in manuscript writing. B.C.: intellectual input into experimental design and interpretation of data and contributed to manuscript writing and review. J.M.: co‐ordinated all author contributions had intellectual input on experimental design and interpretation of data, wrote and edited the manuscript and prepared the final submission. All authors reviewed and approved the final version of the manuscript.

## Disclosure of Potential Conflicts of Interest

B.C. declared commercial royalties from the commercial sales of the antibodies. The other authors indicated no potential conflicts of interest.
